# Promoting Physical Exercise Participation: The Role of Interpersonal Behaviors for Practical Implications

**DOI:** 10.3390/jfmk4020040

**Published:** 2019-06-25

**Authors:** Filipe Rodrigues, Diogo S. Teixeira, Luís Cid, Diogo Monteiro

**Affiliations:** 1 Department of Sports Science, University da Beira Interior (UBI), 6201-001 Covilhã, Portugal; 2 Research Center in Sports, Health Sciences and Human Development (CIDESD), 5001-801 Vila Real, Portugal; 3 Faculty of Physical Education and Sport (ULHT), Lusófona University, Lisboa 1749-024, Portugal; 4 Sport Science School of Rio Maior (ESDRM-IPSantarém), 2040-413 Rio Maior, Portugal

**Keywords:** fitness, motivation, interpersonal behaviors, exercise attendance, exercise adherence

## Abstract

The number of people engaging in physical exercise has been decreasing every year. These behaviors are known to be related with non-communicable chronic diseases and to drastically increase premature morbidity and mortality. Since “the lack of motivation” has been pointed out as one of the main reasons for not engaging in physical exercise, several theoretical and empirical studies have been conducted aimed at understanding what influences behavior regulation. According to literature, gym exercisers who perceive exercise instructors as supportive are more likely to maintain physical exercise participation over the long-run. Supporting autonomy, competence, and relatedness should be carefully considered when interacting with health club clients as a way to promote more autonomous motivation. Overall, it seems that exercise instructors should foster a supportive environment for gym exercisers, in order to encourage exercise as a habitual behavior.

## 1. Introduction

The number of people engaging in physical exercise has been decreasing each year. According to the latest survey from the Physical Activity Council, 82.4 million Americans are physically inactive and indulge in a sedentary lifestyle [1]. These behaviors are known to be related with non-communicable chronic diseases—such as Diabetes Mellitus type 2, hypertension, and hypercholesterolemia—and to drastically increase premature morbidity and mortality [2]. Hence, one wonders why the number of people practicing exercise keeps on decreasing. The fact is that even though it is common knowledge that exercise is extremely efficient when it comes to improving an individual’s health, most of the people fail to engage in exercise practice, pointing out the lack of motivation as the main reason for not practicing any physical activity [3]. The reasons behind this lack of motivation are endless, varying from previous bad experiences at the gym, lack of support by peers, and absence of self-efficacy to act on the behavior itself [4]. In addition, gym and health centers tend to excessively focus on sales and commercial initiatives and goals, bypassing the real nature of physical exercise participation and forgetting that gym exercisers are human beings who want to receive attention and satisfy their physical (i.e., decrease fat loss, increase lean mass), psychological (i.e., increase well-being and enjoyment, decrease depressive symptoms) and social (i.e., relatedness, personal connection) needs [5]. Past studies [6] highlighted the importance that social environment plays on the way exercisers self-regulate their behavior towards physical activity. These statements have been theoretically and empirically confirmed in dozens of studies in the past [4,5], proving that the way someone perceives the external atmosphere impacts self-determined motivation. Therefore, the fitness industry should focus on individual characteristics and consider their needs in order to understand the factors leading to regular exercise participation and avoid withdrawal episodes or complete drop-outs. Understanding why someone engages in physical exercise can alter how fitness professionals interact with them, leading to comprehensive and adaptive behaviors.

However, fitness industry tends to put excessive focus on studies that analyze performance, focusing mainly on performance and physical fitness outcomes [7,8]. Despite scientific interest and encouraging results from these research efforts, such variables have not been highlighted as major contributors when it comes to the increase of physical activity participation rates. This highlights the importance of developing health and exercise-related knowledge for exercise prescription, supported by behavioral theories and strategic facts for exercise promotion [9]. By doing so, not only could exercisers adherence be increased, but also rates of non-commutable chronic diseases could decrease. Therefore, by having an overall understanding of each individual’s situation, the fitness industry could achieve a greater development and consequently reduce public and government spending, especially in health costs. All in all, research on exercise psychology could have an important impact on changing human behavior, since we want to participate in different activities for social bonds, positive emotional outcomes, and motives to drive for self-efficacy and self-determined motivation [10].

Theories concerning human motivation have long been applied to the physical activity context, and recently, multi-theoretical approaches have given us interesting insights. In a recent review conducted by Rodrigues and colleagues [5] grounded in Self-Determination Theory (SDT) [6] and the Hierarchical Model of Intrinsic and Extrinsic Motivation (HMIEM) [11], exercisers perceptions’ of interpersonal behaviors, Basic Psychological Needs (BPN), and self-determined motivation were studied. Results showed the importance of supportive behaviors as forecasters of self-determined motivation, promoting positive emotional, cognitive, and behavioral outcomes. Hence, gym exercisers who perceive exercise instructor as supportive are more likely to maintain physical exercise participation over the long run. Supporting autonomy (i.e., freedom of choice and presentation of alternatives), competence (i.e., positive feedback in specific tasks and encouraging learning) and relatedness (i.e., demonstration of emotional support and concern) should be carefully considered when interacting with gym exercisers. These individuals should be given alternatives to exercises or activities they do not like to perform or have some hardships in terms of execution (i.e., autonomy support). Nevertheless, exercisers should feel they are engaging in challenging training programs to improve their skills and acquire new abilities, and receiving positive and constructive feedback when performing specific exercises in order to avoid feelings of doubt or low perception of self-efficacy (i.e., competence support). Lastly, exercisers should experience sensitive support, care and emotional provision from the exercise professional, ensuring relationships of trust and to feel connected (i.e., supporting relatedness). Since BPNs have been related to higher levels of exercise persistence and are highly influenced by the social context [5], it seems that gym personnel are accountable for improving individuals’ experience of exercising. The satisfaction of BPN has been intensively associated with more self-determined behavior, which in turn may explain why the gym exercisers acts on the behavior because of pleasure and enjoyment, without expecting external rewards or pressures by significant others (i.e., peers, family members, or fitness instructors). Endorsing in supportive interpersonal behaviors has been shown to promote exercisers’ BPN, self-determined motivation, and commitment, and facilitates continuous exercise adherence [12].

Research tracking gym adherence found that drop-out rates reach the 75% mark after the first three months and 50% after the six months of exercise practice [13]. Thus, one could forecast past exercise frequency to be a predictor of future exercise commitment, and that a regular exercise attendance in gym activities in the early stages may increase the possibility of maintaining the behavior over the long-run. However, studies focusing on habitual behaviors have shown that even complex behaviors such as physical activity (e.g., programming weekly sessions, packing sports bag, following exercise instructors training schedule, going to the gym, and so forth) can turn into a low-conscious and low mental effort routine [14]. This automaticity assumes an extremely important role for long-term exercise practice, showing that the behavior could be anchored in one’s daily routine. Should this fail to happen, sooner or later, people usually end up by dropping-out exercise participation.

Past positive experiences regarding exercise participation might increase the chances of someone maintaining the behavior in the future [15,16]. For example, if the gym exerciser experiences support by exercise instructors, recognizes fitness group classes as fun activities, and feels self-efficacy when executing exercises, then the degree of persistence in the behavior increases. According to theoretical frameworks, higher intentions to perform a specific behavior (in this case exercise) are related to higher likelihood of performing the behavior itself [17]. In other words, when an individual perceives factors that may facilitate the operationalization of the behavior (e.g., leaving gym clothes and mp3 in the bag) and has individual beliefs that he has ease to perform the behavior (e.g., scheduling training sessions), then it is more likely that he will endorse regularly in the behavior; past studies help to sustain this point of view [12,15]. In fact, studies [18,19] have shown that outcomes such as enjoyment could be a strong predictor of intention in the context of sports. These studies do not stand alone, since other researchers have reached similar results, showing the impact professionals’ (e.g., coaches, instructor, and teachers) behavior has on others [20].

## 2. Recommendations to Apply

- Endorse in supportive behaviors when interacting with gym exercisers. Do not use negative feedback, nor impose pressure to perform only pre-programmed exercises. Instead, encourage to improve technique and give exercisers some volitional choice on which exercises to do, that are aligned with the main structure of the individual exercise prescription.
- Create conditions for fun and pleasurable climates. During gym activities, understand what makes clients laugh, and learn more about them to promote enjoyable atmosphere with others.
- Look at gym exercisers as emotional human beings and not just as a “membership numbers”. Making money is crucial in the fitness industry, but considering the big picture, gyms and health centers rely on client retention. Personal training and other services are an upselling increase in the fitness industries’ income. Therefore, if exercisers do not perceive fitness instructors as trustworthy and emotionally connected, then the likelihood to drop out is almost guaranteed.
- Generate effective questions during training session in order to be perceived as someone attentive and regardful professional: “what are the exercises you enjoy the most?”; “what factors do you feel may have contributed to your weight loss/gain?”; “what do you experience when enrolling in high intensity training sessions?” or “what activities do you feel most competent?” are examples of open-ended questions exercise instructors should use during training session to understand the emotional state exercisers experience when exercising with them.

## 3. Overall Considerations

The take-home message highlights the role that exercise instructor have, and should actively pursue in order to promote and enable physical exercise behavior change in gym exercisers. Fitness professionals’ interpersonal behaviors and communication are means to success, by gaining clients respect and confidence. Overall, it seems that exercise instructors should foster a supportive environment for gym exercisers in order to promote exercise as a habitual behavior and be a part of the exercisers’ life. Additionally, positive feedback should be given, and clients should have the chance to select the exercises or activities they appreciate to perform in alignment with individual goals and evidence-based prescription. Moreover, promoting empathy and social support seems to be important for stimulating more self-determined motivation. In addition, exercise needs to be prescribed as a fun and relatable activity, and not only as a “biomechanical straightforward” plan. Exercisers are emotional human beings; therefore, social interactions play a major role when it comes to affective bonding with fitness professionals. Taking everything into account, one should bear in mind that the social environment (i.e., gym managers, technical coordinators, and exercise instructors) is an important predictor of exercisers’ self-determined motivation, which is fundamental to maintain exercise participation.

Professional scientific and pedagogical development should continue to focus in biomechanics, physiology, and other relatable health and sport fields of study, aiming to provide a continuous improvement in evidence-based exercise prescription; but this should be supported by health behavior change techniques based on robust theoretical frameworks to increase exerciser persistence and adherence.

## Abbreviations

SDT	Self-Determination Theory
HMIEM	Hierarchical Model of Intrinsic and Extrinsic Motivation
BPN	Basic Psychological Needs
